# A Novel Strategy for Preparation of Si-HA Coatings on C/C Composites by Chemical Liquid Vaporization Deposition/Hydrothermal Treatments

**DOI:** 10.1038/srep31309

**Published:** 2016-08-05

**Authors:** Xiong Xin-bo, Ni Xin-ye, Li Ya-yun, Chu Cen-cen, Zou Ji-zhao, Zeng Xie-rong

**Affiliations:** 1 Shenzhen key laboratory of special functional materials, Shenzhen engineering laborary for advanced technology of ceramics, Department of materials science and engineering, Shenzhen University, Shenzhen 518060, China; 2 Second People’s Hospital of Changzhou, Nanjing Medical University, Changzhou 213003, China

## Abstract

A novel strategy for the preparation of Si-doped hydroxyapatite (Si-HA) coatings on H_2_O_2_-treated carbon/carbon composites (C/C) was developed. HA coating was prepared on C/C through chemical liquid vaporization deposition (CLVD)/hydrothermal treatment. HA coating was immersed in an H_2_SiO_3_ solution at an autoclave at 413 K for transformation into Si-HA coating. The effects of H_2_SiO_3_ mass contents on the phase, morphology, and composition of the Si-HA coatings were studied through SEM, EDS,XRD, and FTIR. Their bonding performance to C/C was measured through a scratch test. Under the optimal content condition, the *in vitro* skull osteoblast response behaviors of the Si-HA coating were evaluated. Results showed that SiO_3_^2−^ could enter into the HA lattice and occupy the PO_4_^3−^ sites. Doped SiO_3_^2−^ significantly improved the bonding performance of the HA coating to C/C in comparison with the untreated HA. The adhesive strength of the coatings initially increased and then decreased with increasing H_2_SiO_3_ content. Meanwhile, the cohesive strength of the Si-HA coatings was almost nearly identical. The Si-HA coating achieved at a content of 90% H_2_SiO_3_ exhibited the best bonding performance, and its osteoblast compatibility *in vitro* was superior to that of the untreated HA coating on C/C through CLVD/hydrothermal treatment.

Carbon is a vital element in the human body. It often exists as a compound *in vivo*. Furthermore, carbon is often extensively explored as a biomedical material in the form of a single substrate, such as pyrolic carbon, diamond-like carbon, and carbon fiber, because of its excellent biocompatibility^1^. However, its brittleness limits its further applications in orthopedics. Its composites, such as carbon fiber enhancing carbon matrix(C/C), not only maintain the bio-inertness of carbon materials but also possess good strength and pseudo ductility. In particular, they possess low rigidity similar to that of the human cortical bone, thereby avoiding the stress-shielding effects; thus, they are regarded as a novel generation of biomedical composites in artificial joint and bone prosthetics^2^,^3^. However, untreated C/C composites have a hydrophobic surface and show no biological activity^4^. After implantation *in vivo*, they show no function of the conduction or induction of bone tissue regeneration and cannot chemically bond with human tissues. Moreover, the fixation cycle of C/C implants *in vivo* requires relatively more time than that of other bone materials because of the weak connection between C/C and close tissues. Their applications as bone replacement and repair materials are thus affected^5^.

Hydroxyapatite (HA), with a chemical formula of Ca_10_(PO_4_)_6_(OH)_2_, extensively exists in the bone tissues and teeth of animals^6^. Despite being a bio-active material with good bonding strength to bone tissues, HA cannot be applied as a bone implant alone because of its shortcomings in strength and toughness^7^. Therefore, combining the advantages of C/C composites with those of HA is a logical choice to fulfill the requirements of human body implants in bone replacement; C/C composites are utilized as the matrix and HA as a surface coating^8^,^9^. However, pure HA easily dissolves in physiological environments, and its instability often causes implantation failure^10^. Furthermore, the biological activity of pure HA is inferior to that of real bone minerals^11^ and thus needs to be improved.

Silicon (Si) is a benignant trace element in human bone tissues, and its ion form, silicate, directly affects bone growth and development^12^. Silicate can infiltrate into all sites during the growth of human bones, and its content is relatively high in the early calcification period of the bone matrix, thereby demonstrating its enhanced HA induction ability in human tissues^13^. The experiment conducted by Thian *et al.* demonstrated that the percentage of bone growth on Si-HA coating is significantly larger than that on pure HA^14^. As demonstrated by several studies, the lack of silicate restricts animal growth and induces bone dysplasia and deformation and dental enamel dysplasia^15^. The *in vitro* studies conducted by Sun *et al.* showed that the silicate ion promotes cell proliferation and differentiation by shortening the cell division cycle. It can alsoup-regulate the expression of osteoblast differentiation genes, such as BMP-2, ALP, and Runx2, to stimulate the differentiation and mineralization of osteoblasts. In addition, the silicate ion can up-regulate the expression of the OPG gene, down-regulate the expression of the RANKL gene, and inhibit osteoclast activity^16^,^17^. Therefore, HA could be doped with the silicate ion to further improve its bio-performance.

Several methods, such as magnetron co-sputtering^18^, hydrothermal technology^19^, plasma spraying^20^, pulsed laser deposition^21^, aerosol deposition^22^, electrochemical methods^23^, and biomimetic deposition^24^, have been applied to prepare Si-doped HA (Si-HA) coatings. However, most of these technologies have been applied to titanium substrates. Only the ultrasound-assisted electrochemical deposition method has been adopted to deposit Si- and Na-doped HA onto C/C composites; the method has been reported to demonstrate only the induction ability of an apatite layer *in vitro*^4^. To our knowledge, no public report has been presented with regard to hydrothermal treatment for the preparation of Si-HA coating on C/C or other substrates and the simultaneous adhesion and bio-compatibility of Si-HA coatings on C/C. In our previous studies, the chemical liquid vaporization deposition method (CLVD) was introduced to deposit calcium phosphate coating, including HA, onto C/C with extremely high adhesive strength^25^. This method possesses many advantages, such as thin films, good control over the deposited solid phase, and short processing time, and demonstrates a promising prospect in orthopedics. To enhance the performance of pure HA on C/C as a biomedical material, we developed a novel strategy for the preparation of Si-HA coating on C/C. Specifically, HA coating was prepared through CLVD with post-hydrothermal treatment and then converted to Si-doped HA coating by hydrothermal treatment in H_2_SiO_3_ solution. The effects of H_2_SiO_3_ contents on the composition, microstructure, bonding performance, and *in vitro* cell response of the Si-HA coatings were studied in detail.

## Materials and Methods

### Preparation of Si-doped HA coatings with C/C composites

C/C samples with a size of 10 × 10 × 10 mm^3^, provided by Shanghai University were repeatedly cleaned with distilled water and anhydrous alcohol. Subsequently, the C/C samples were placed in a 50 mL autoclave with 2 M H_2_O_2_ solution for hydrothermal treatment at 413 K for 4 h to obtain a hydrophilic surface. After removal, the samples were ultrasonically washed with distilled water and then air dried for further use. Afterward, a deposition aqueous solution with 0.2 M NH_4_H_2_PO_4_ and 0.6 M Ca(NO_3_)_2_ was utilized to deposit monetite coatings on C/C by CLVD. The experimental equipment has been described in another study^26^, and its working frequency was set to 330 kHz. The monetite-coating C/C samples were then immersed in an autoclave with 10% ammonia solution and treated at 413 K for 12 h to obtain pure HA coatings. Lastly, the HA coatings were further hydrothermally treated in an aqueous solution with 80%, 85%, 90%, and 95%(mass percentage) silicic acid as fillers. After the treatment, the samples were removed, ultrasonically washed with distilled water, and dried with a hairdryer for using in the following characterizations.

### Material characterization

A D8 Advance X-ray diffractometer (XRD;Bruker-Axs Co., Karlsuuhe, Germany) (Cu-Ka radiation) was employed to characterize the phases of the powder scrapped from the coating C/C samples. The morphology and composition of the samples were characterized through scanning electron microscopy (SEM) and energy dispersive spectroscopy (EDS) (S-3400N, Hitachi High Technologies Co., Tokyo, Japan). An FTIR-8300PCS Fourier infrared spectrometer (FTIR; Perkin Elmer Co., Norwalk, America) and KBr pellet technology were used to quantitatively identify the chemical functional groups of the coatings. The FTIR spectra was recorded at the 400–4000 cm^−1^ range and at a resolution of 4 cm^−1^. The chemical state of the Siatom in the H_2_SiO_3_ hydrothermally treated HA coating was investigated through X-ray photoelectron spectroscopy (XPS; ULVAC-PHI 1800, Japan) with Al Kα X-ray source (1486.6 eV). Prior to the XPS measurement, the coating C/C sample was etched using an argon gun with its energy being 4 Kev, current equal to 1 μA and the errosion time close to 1200 s. To calibrate every spectrum, the binding energy of the C1s level from contamination of saturated hydrocarbons at 284.60 eV was used as an internal reference. The adhesive force of the coating to the C/C substrate was determined through a scratch test by using an s-3400N scratch tester (CSM, Switzerland) fitted with a Rochwell C 0.2 mm diamond stylus with a preload of 1N, load speed of 120 N min^−1^, scratch speed of 5 mm min^−1^, and maximum load of 120 N.

### Cell proliferation experiments

The cell proliferation experiments were conducted on the coatings with MTT colorimetric method. The human skull cells utilized in the experiments were provided by Nanjing Medical University. A 24-hole culture plate was utilized to place the pure and Si-HA coating samples and subsequently added to RPMI1640 and DMEM solutions. After soaking for 24 h, the cells with a density of 2 × 10^4^/cm^2^ were added and inoculated onto the sample surfaces and cultured again in an incubator with 5% CO_2_ at 310 K. During the experiments, three groups of the coating samples were compared. Each group had four samples, among which the three samples were utilized for cell proliferation characterizations and the rest for cell morphology observation. After 2, 4, and 6 d of inoculation, 200 μL of MTT agents at 5 mg/ml were added for MTT detection. The absorbance A value was detected with a microplate reader at a wavelength of 570/630 nm. The morphology and adhesion features of the cells were observed through SEM, and the proliferation data were statistically analyzed through ANOVA with SPSS11.0 software.

The alkaline phosphatase (ALP) activities of the HA and optimal Si-HA coatings were evaluated through bi-antibody single-step ELISA. The skull cell inoculation process of the coatings was similar to that in the cell proliferation test. Each group has three pieces of the test samples. After cell inoculation for 2, 4, 6, and 8 d, 0.25 g/L pancreatin was added to digest the samples. The resulting digestive juice was then centrifuged to eliminate the supernatant. As a result, the cell suspension was obtained and transferred to a 96-hole culture plate. Subsequently, the cell suspension in the holes of the culture plate holes was added to ALP oligomer and cultured for 30 min at 310 K. The optical density of each hole was measured with an enzyme-linked detector for ALP quantification at 410 nm at different culture durations.

## Results and Discussion

### XRD analysis of coatings

**Figure 1:**
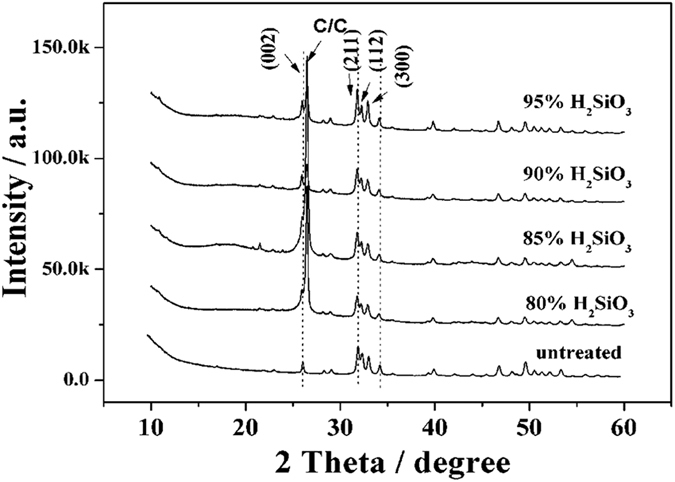
XRD patterns of as-achieved coatings at different H_2_SiO_3_ contents at 413 K.

Figure 1 shows the XRD patterns of the coatings achieved at 413 K at different H_2_SiO_3_ contents. Aside from a carbon peak, the coating prepared by CLVD followed by hydrothermal treatment presented an evident HA structure with three characteristic peaks of (211), (112), and (300) planes^27^. After the hydrothermal treatment, the as-achieved coatings still showed the same characteristic peaks as the HA coating, indicating that no impurity phase was produced except for HA after H_2_SiO_3_ treatment. However, compared with the untreated HA, the HA treated by H_2_SiO_3_ exhibited a slight shift to a low diffraction degree in the (002) and (300) peaks, which became increasingly defined with the increase in H_2_SiO_3_ contents. This condition indicates an increase in the interplanar spacing of the (002) and (300) crystal planes. The H_2_SiO_3_ hydrothermal treatment caused the elongation of a and c axes in the close-packed hexagonal lattice of HA, suggesting the successful infiltration of SiO_4_^4−^ into the HA lattice and the acquisition of Si-doped HA (Si-HA). In the Si-HA structure, the PO_4_^3−^ sites are often partially substituted by SiO_4_^4−^. The radius of Si^4+^ (0.042 nm) is larger than that of P^5+^ (0.035 nm), and the length of the Si-O bond (0.161 nm) is larger than that of the P-O bond (0.155 nm)^24^, which resulted in the increase in the lattice parameters.

### Analysis of the morphology and chemical composition of coatings

**Figure 2:**
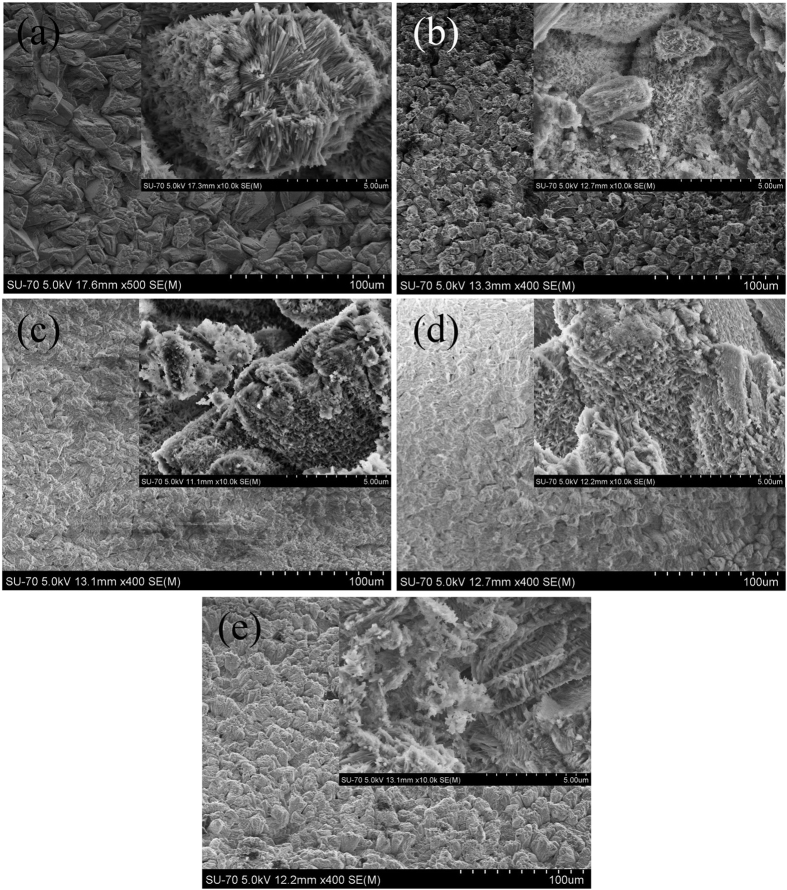
Morphologies of coatings hydrothermally treated by H_2_SiO_3_ at different concentrations at 413 K: (**a**) untreated; (**b**) 80%; (**c**) 85%; (**d**) 90%; and (**e**) 95%.

Figure 2 shows the morphologies of the pure HA and hydrothermally treated coatings with different contents of H_2_SiO_3_ as fillers at 413 K. For the untreated HA coating, large particles were observed on the top view SEM image (Fig. 2(a)). However, for the Si-HA coatings, only small particles existed, which constructed a much denser surface than that of the pure HA coating. The morphology changes of the coatings indicate that the formation of the Si-HA coatings experienced dissolution and recrystallization. Moreover, as the H_2_SiO_3_ contents increased, the compactness of the coating initially increased and then decreased. At 90% H_2_SiO_3_ content, the Si-HA surface showed the highest compactness because the high silicic acid content hampered the adequate dissolution of the HA crystals and resulted in the incomplete solution of initial HA crystals and a porous coating morphology. Images magnified by 10,000 times are shown in the insets of Fig. 2(a–e). The surface of the untreated HA coating was composed of distributed nano-needle-like particles and full holes between particles and displayed a loose morphology. After hydrothermal treatment in the silicic acid solution, the size of the particles decreased and they became closely linked, thereby constructing a relatively compacter surface than that of the untreated HA coating. Additionally, no obvious difference was observed in the morphologies of the Si-HA coatings.

**Figure 3:**
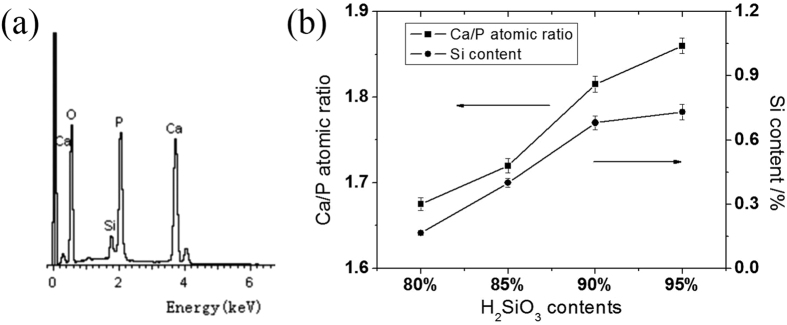
A typical energy spectrum of a Si-HA coating (**a**) and the dependence curve of Ca/P atomic ratio and Si content of Si-HA coatings (**b**).

A typical EDS spectrum is presented in Fig. 3(a). The figure shows the presence of Si, except for Ca, P, and O, in the HA phases, further verifying the presence of Si in the HA coating. The untreated HA coating has a Ca/P atomic ratio of 1.57, which is lower than the theoretical value of HA. The dependence curve of the coating compositions on the silicic acid contents after doping Si to HA is shown in Fig. 3(b). With the increase in silicic acid contents, the Si content and Ca/P atomic ratio of the Si-HA coatings gradually increased and were all larger than those of the treated HA. This result is due to the replacement of PO_4_^3−^ by SiO_4_^4−^, leading to a decrease in the P content of HA.

### FTIR and XPS analyses of coatings

**Figure 4:**
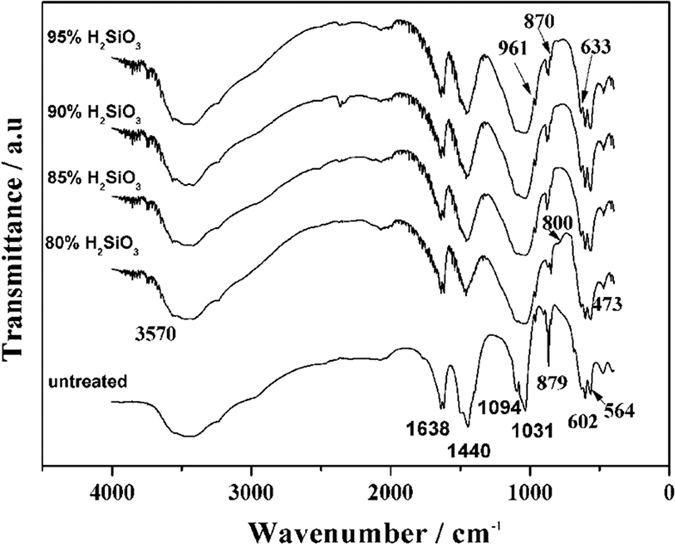
FTIR spectra of untreated and Si-HA coatings.

**Figure 5:**
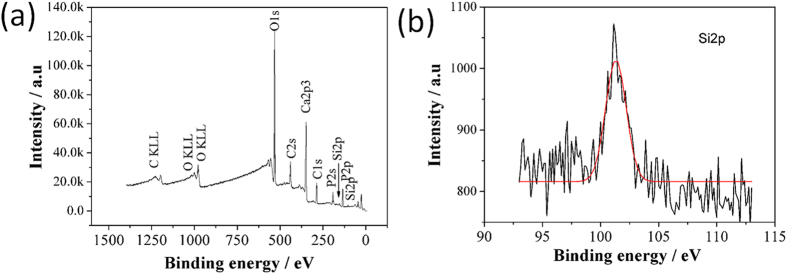
XPS profiles of the HA coating on C/C treated by a 80% H_2_SiO_3_ solution (**a**) survey scan; (**b**) Si2p narrow scan.

Figure 4 presents the FTIR spectra of the HA and Si-HA coatings. The peaks at 3570 and 633 cm^−1^ are ascribed to the absorption peak of OH^−^ caused by the stretching vibration of OH^−^. The peak at 1638 cm^−1^ is resulted from water^28^. The peaks at 564, 602, 1031, and 1094 cm^−1^ denote the absorption peaks of PO_4_^3−^. The peaks at 1440 and 879 cm^−1^ are assigned to the absorption peaks of CO_3_^2−^, and the peak at 1440 cm^−1^ is the stretching vibration peak of CO_3_^2−^ resultingfrom its partial occupation of the sites of PO_4_^3−^, this indicates the achievement of B-type HA coatings^29^. With the increase in H_2_SiO_3_ contents, the two peaks of PO_4_^3−^ at 1031 and 1094 cm^−1^ overlapped each other because the SiO_3_^2−^ groups infiltrated into the HA lattice and replaced a fraction of PO_4_^3−^ ions^30^. Meanwhile, a weak peak at 800 cm^−1^ was observed and ascribed to the vibration absorption peak of Si-O^12^. In addition, with the increase in H_2_SiO_3_ contents, the relative intensity of the hydroxyl peak at 633 cm^−1^ in the Si-HA coatings also increased as a result of the displacement of PO_4_^3−^ by SiO_4_^4−^. Si substituted for P sites in the PO_4_^3−^of the HA lattice, leading to an increase in the positive charge of HA. Thus, more hydroxyl ions were absorbed onto HA to maintain the charge balance. Consequently, the resulting OH^−^ contents increased in the Si-HA coatings. The XPS spectra of the HA coating on C/C treated by 80% H_2_SiO_3_ are shown in Fig. 5(a,b). The full spectrum shows that the coating contained O, P, and Si elements, and its Ca/P atomic ratio and Si content are 1.58 and 0.5%, respectively. The Si2p binding energy ocurred at 101.1 eV, which is similar to that in the Si atoms in an ortho-silicate group^31^ rather than a metasilicate group, also proving the successful incorporation of Si into the HA lattice.

### Scratch tests of coatings

**Figure 6:**
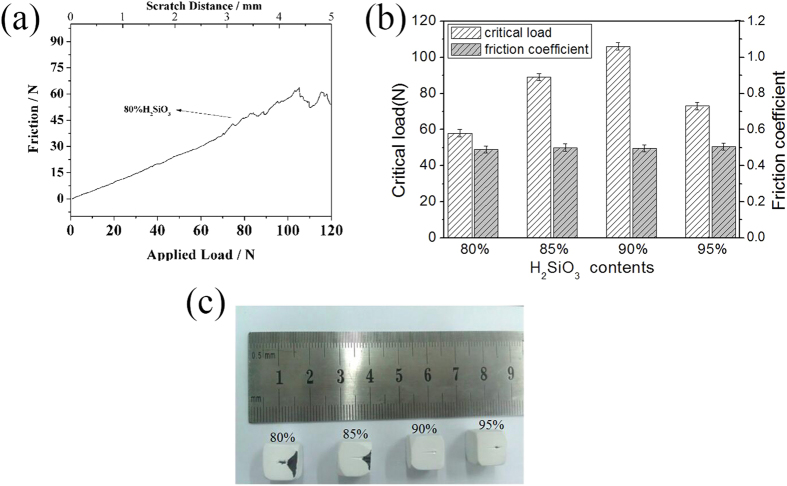
A typical curve of friction force vs applied load/scratch distance (**a**), Comparisons of critical load/friction coefficient of Si-HA coating at different H_2_SiO_3_ contents (**b**) and Failure morphologies of Si-HA coatings on C/C (c).

The adhesive strength of the coating on C/C was characterized through a scratch test. Figure 6(a) shows a typical scratch curve of the Si-HA coating achieved at 80% silicic acid content. The dependence of the applied vertical load on friction force/scratch distance was reflected. Together with the failure morphology observation obtained by an optical microscope attached to the scratch tester, the critical load and friction force of the coating on the C/C substrate can be determined. The friction coefficient can then be obtained through the friction law.


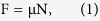


where F is the friction force, μ is the friction coefficient, and N is the applied load. In light of the achieved friction force and coefficient, the adhesive and cohesive strengths of the coatings on substrates can also be calculated. The relation of the critical load and friction coefficient of the coating to the H_2_SiO_3_ content is presented in Fig. 6(b). The critical load of the Si-HA coatings was 58, 89, 106, and 73N at H_2_SiO_3_ contents of 80%, 85%, 90%, and 95%, respectively. These values are higher than that of the untreated HA coating(47N)^25^. Meanwhile, the friction coefficient of all the Si-HA coatings was almost the same and reached a mean value of 0.5, which is larger than that of the pure HA coating (0.22). In a scratch test, the critical load reflects the adherent strength of coating^32^, while the friction coefficient is the embodiment of the close cohesive strength of the two coatings’ crystals^33^. Thus, we can deduce that SiO43- infiltration into the HA lattice could increase the cohesive and adhesive strengths of the HA coating. The cohesive strength enhancement of the Si-HA coating is due to the strengthening effect of doped SiO_4_^4−^ ions in HA lattice. Whereas, the adhesion strength improvement of the Si-HA coating might be ascribed to the bonding of the Si-HA to H_2_O_2_-treated C/C substrate through the formation of Si-O-O-C=O or Si-O-H∙∙ O=C composite structure. This is because, during the coating preparation, only parts of the H_2_O_2_-treated C/C surface were covered with HA. After the hydrothermal silicic acid treatment, the as-deposited CaHPO_4_ would dissolve. In the process that the disolved HA further converted to the Si-HA, the Si-HA could be connected to the uncovered C/C surface. Meantime, when the HA dissolved, the coating would leave some voids, which also facilitate silicic acid groups diffuse into and fixed onto the C/C surface. Therefore, there would be more the C/C surface connecting with the Si-HA coating, thus increasing the adhesive strength of the coating. The failure morphologies of the Si-HA coatings are shown in Fig. 6(c). The Si-HA coatings obtained at 80% and 85% contents of silicic acid showed a “peeling off” from the C/C substrate after they failed; they presented a catastrophic damage mode. By contrast, the coatings achieved at 90% and 95% silicic acid contents presented well-defined scratch traces. In particular, at 90% silicic acid content, no obvious collapsing fragments occurred despite the coating failure. This condition means that the Si-HA coating hydrothermally treated by silicic acid has the best plastic deformation behavior among all the HA coatings. From these analyses, we conclude that 90% content of silicic acid is one of the optimal processing parameters for the treatment of HA coating through CLVD followed by hydrothermal treatment to obtain Si-HA coating on C/C. The Si-HA coating obtained at this silicic acid content was then applied to evaluate its cell compatibility *in vitro*.

### Cell biocompatibility of untreated and treated coatings *in vitro*

**Figure 7:**
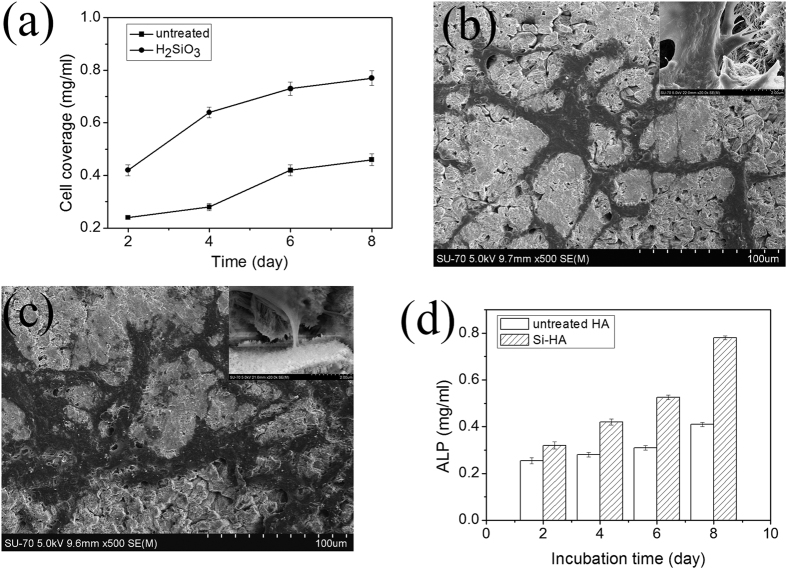
Comparisons of cell coverages (**a**), cell morphologies on the untreated HA coating (**b**) and Si-HA coating (**c**), ALP activities (**d**) between untreated and Si-HA coatings achieved at a 90% H_2_SiO_3_ content.

Figure 7(a) shows the proliferation curves of human skull osteoblasts inoculated into the HA and Si-HA coatings on C/C at 2, 4, 6, and 8 d. The Si-HA coating showed markedly improved cell proliferation (exhibited good cell proliferation)when compared with the untreated HA coating. The P values at 2, 4, 6, and 8 d obtained through ANOVA were 0.1 × 10^−4^, 7.5 × 10^−6^, 5.4 × 10^−6^, and 6.7 × 10^−6^, respectively. These values are lower than 5.0 × 10^−2^, indicating that the proliferation difference of the coatings is statistically significant. The surface roughness variation may also affect cellular responses^34^, but in a certain range, roughness change (3.36 μm down to 0.13 μm), the osteoblast proliferation were found to be insignificant^35^.

The morphologies of the osteoblasts on the HA and Si-HA coatings cultured for 2 d are shown in Fig. 7(b,c). The osteoblasts could cover and adhered tightly to the coating surfaces. The cell covered area of the Si-HA coating was larger than that of the HA coating on C/C, revealing the larger cell numbers of the Si-HA coating. A magnified photo of the osteoblasts on the Si-HA coating shows that their pseudopods adhered tightly to crystals in the coating. These results further demonstrate the superior cell compatibility of the Si-HA coating on C/C. The ALP results also show that the values of the Si-HA coating are higher than that of the untreated HA coating (Fig. 7(d)). This result further proves the high osteoblastic activity of the Si-HA coating.

The Si-doped HA coatings can increase the biological activity of implants *in vitro*, which may be related to the changes in the HA structure and could result in a potential drop. The silicate groups substitute for parts of phosphate groups, thereby endowing HA with negative charges. Consequently, the HA surface absorbs more protons, making it possess more abundant hydroxyl groups, as revealed by FTIR. This enhances the hydrophilic property of HA. The more hydrophilic the surface of biomedical materials is, the better their cell biocompatibility is^36^. Thus, owing to improved wet-ability, the Si-HA surface can easily facilitate the adsorption and adhesion of osteoblasts, leading to osteogenic growth and proliferation.

From these analyses, we conclude that hydrothermal treatment with an H_2_SiO_3_ solution offers a strong potential route for HA improvement in orthopedic and dental applications.

## Conclusions

We proposed a strategy for the preparation of Si-doped HA coatings on C/C composites by hydrothermally treating HA through CLVD/hydrothermal method in an H_2_SiO_3_ solution at 413 K. With this strategy, Si successfully infiltrated into the HA lattice. Si doping significantly enhanced the bonding performance of the HA coatings. With the increase in H_2_SiO_3_ mass contents, the critical load of the Si-HA coatings on C/C initially increased and then decreased. The optimal mass content of H_2_SiO_3_ to achieve the best bonding performance of Si-HA coating to C/C was 90%. The Si-HA coating on C/C achieved under the optimal content exhibited remarkably improved cell proliferation ability in comparison with the untreated HA coating.

## Additional Information

**How to cite this article**: Xin-bo, X. *et al.* A Novel Strategy for Preparation of Si-HA Coatings on C/C Composites by Chemical Liquid Vaporization Deposition/Hydrothermal Treatments. *Sci. Rep.* **6**, 31309; doi: 10.1038/srep31309 (2016).
